# Draft genome of *Rhodococcus* sp. no. 34, a soil isolate capable of curcumin degradation

**DOI:** 10.1128/mra.01249-25

**Published:** 2026-01-28

**Authors:** Yoshiteru Hashimoto, Kana Ishigami, Takuto Kumano, Michihiko Kobayashi

**Affiliations:** 1 Institute of Applied Biochemistry and Graduate School of Life and Environmental Sciences, University of Tsukuba 98393, Tsukuba, Ibaraki, Japan; 2 Microbiology Research Center for Sustainability (MiCS), University of Tsukuba 623473, Tsukuba, Ibaraki, Japan; 3 Tsukuba Institute for Advanced Research (TIAR), University of Tsukuba 13121, Tsukuba, Ibaraki, Japan; 4 Center for Quantum and Information LifeSciences, University of Tsukuba 13121, Tsukuba, Ibaraki, Japan; University of Manitoba, Winnipeg, Canada

**Keywords:** curcumin metabolism, soil bacterium, actinomycete

## Abstract

We report the draft genome sequence of *Rhodococcus* sp. no. 34, which degrades curcumin, the active ingredient in turmeric. The assembled genome consists of 51 contigs totaling 6,364,057 bp, with an *N*_50_ of 733,884 bp and a G + C content of 62.5%.

## ANNOUNCEMENT

Previously, we reported the isolation of *Rhodococcus* sp. no. 34 from soil collected at Mt. Tsukuba, Ibaraki, Japan (36.23°N, 140.10°E) using a screening medium containing curcumin, a yellow polyphenolic compound with antioxidant and anti-inflammatory properties, as the sole carbon source (1). From this strain, we purified a curcuminoid hydrolase (named CurH), which is presumed to play a physiological role in curcumin metabolism, and identified the structural gene for CurH acting on natural curcuminoids. To date, no genome sequence of a curcumin-degrading microorganism has been reported.

Strain no. 34 was cultivated in 2× YT medium (2) at 28°C for 48 h. Cells were frozen in liquid nitrogen and crushed using a pestle and mortar. Genomic DNA was extracted using Marmur’s method (3). Extracted DNA was treated with a phenol solution (4) and RNase, and then purified by ultracentrifugation (2). A 350 bp insert library was prepared using the TruSeq Nano DNA Library Prep Kit (Illumina, San Diego, CA, USA) and sequenced on an Illumina HiSeq 2500 platform, yielding 44,105,954 paired-end reads (100 bp, 4.455 Gb total). Adapter removal and quality trimming were performed using Cutadapt v1.1 (5) and Trimmomatic v0.32 (6), respectively. The genome was assembled with Velvet v1.2.08 (7), and genome annotation was performed using DFAST v1.3.6 (https://dfast.ddbj.nig.ac.jp) with default parameters (8). Genome completeness was assessed with BUSCO v6.0.0 (9) in genome mode using the actinomycetota_odb12 data set (*n* = 238). Default parameters were used unless otherwise noted.

The assembled genome of strain no. 34 consists of 51 contigs, with 693× coverage and an *N*_50_ value of 733,884 bp. The draft genome totals 6,364,057 bp and has a G + C content of 62.5%. BUSCO analysis showed 97.1% completeness (96.2% single-copy, 0.8% duplicated), 2.1% fragmented, and 0.8% missing. A total of 5,893 protein-coding genes, 57 tRNAs, and 2 rRNAs were predicted.

The genome sequence of strain no. 34 will provide a valuable resource for elucidating bacterial curcumin metabolism in the natural rhizosphere.

## Data Availability

This Whole Genome Shotgun project has been deposited in DDBJ/ENA/GenBank under accession numbers BAAILK010000001–BAAILK010000051. The raw sequencing reads are available in the DDBJ Sequence Read Archive under accession number DRR786785 (BioProject number PRJDB37580 and BioSample number SAMD01687583).

## References

[1] Hashimoto Y, Ishigami K, Hassaninasab A, Kishi K, Kumano T, Kobayashi M. 2024. Curcumin degradation in a soil microorganism: screening and characterization of a β-diketone hydrolase. J Biol Chem 300:107647. doi:10.1016/j.jbc.2024.107647 39122010 PMC11407993

[2] Sambrook J, Russel D. 2001. Molecular cloning: a laboratory manual. 3rd ed. Cold Spring Harbor Laboratory Press, Cold Spring Harbor, NY.

[3] Marmur J. 1961. A procedure for the isolation of deoxyribonucleic acid from micro-organisms. J Mol Biol 3:208–IN1. doi:10.1016/S0022-2836(61)80047-8

[4] Hopwood DA, Bibb MJ, Chater KF, Kieser T, Bruton CJ, Kieser HM, Lydiate DJ, Smith CP, Ward JM, Schrempf H. 1985. Genetic manipulation of Streptomyces: a laboratory manual. John Innes Foundation, Norwich, UK.

[5] Martin M. 2011. Cutadapt removes adapter sequences from high-throughput sequencing reads. EMBnet J 17:10. doi:10.14806/ej.17.1.200

[6] Bolger AM, Lohse M, Usadel B. 2014. Trimmomatic: a flexible trimmer for Illumina sequence data. Bioinformatics 30:2114–2120. doi:10.1093/bioinformatics/btu170 24695404 PMC4103590

[7] Zerbino DR, Birney E. 2008. Velvet: algorithms for de novo short read assembly using de Bruijn graphs. Genome Res 18:821–829. doi:10.1101/gr.074492.107 18349386 PMC2336801

[8] Tanizawa Y, Fujisawa T, Nakamura Y. 2018. DFAST: a flexible prokaryotic genome annotation pipeline for faster genome publication. Bioinformatics 34:1037–1039. doi:10.1093/bioinformatics/btx713 29106469 PMC5860143

[9] Manni M, Berkeley MR, Seppey M, Simão FA, Zdobnov EM. 2021. BUSCO update: Novel and streamlined workflows along with broader and deeper phylogenetic coverage for scoring of eukaryotic, prokaryotic, and viral genomes. Mol Biol Evol 38:4647–4654. doi:10.1093/molbev/msab199 34320186 PMC8476166

